# Stereochemical Editing at sp^3^-Hybridized Carbon Centers by Reversible, Photochemically Triggered Hydrogen Atom Transfer

**DOI:** 10.1021/acs.accounts.4c00830

**Published:** 2025-02-19

**Authors:** Maximilian Iglhaut, Thorsten Bach

**Affiliations:** Department of Chemistry and Catalysis Research Center (CRC), Technical University of Munich, Lichtenbergstr. 4, 85747 Garching, Germany

## Abstract

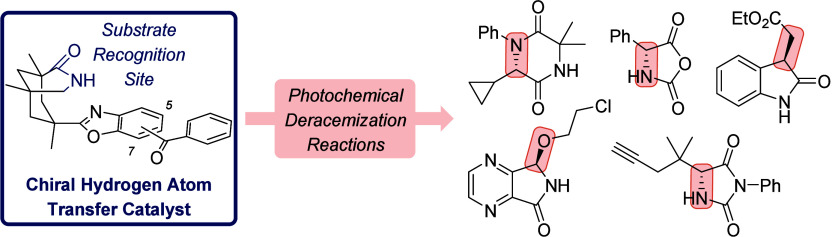

Millions of chiral compounds contain a stereogenic sp^3^-hybridized carbon center with a hydrogen atom as one of the four different substituents. The stereogenic center can be edited in an increasing number of cases by selective hydrogen atom transfer (HAT) to and from a photocatalyst. This Account describes the development of photochemical deracemization reactions using chiral oxazole-annulated benzophenones with a bonding motif that allows them to recognize chiral lactam substrates by two-point hydrogen bonding. The backbone of the catalysts consists of a chiral azabicyclo[3.3.1]nonan-2-one with a U-shaped geometry, which enables substrate recognition to occur parallel to the benzoxazole part of the aromatic ketones. The photocatalysts facilitate a catalytic photochemical deracemization of several compound classes including hydantoins, *N*-carboxyanhydrides, oxindoles, 2,5-diketopiperazines, and 4,7-diaza-1-isoindolinones. In addition, if more than one stereogenic center is present, the editing delivers a distinct diastereoisomer upon the appropriate selection of the respective photocatalyst enantiomer. The chiral photocatalysts operate via the benzophenone triplet that selectively abstracts a properly positioned hydrogen atom in exclusively one of the two substrate enantiomers. The photochemical step creates a planar carbon-centered radical and erases the absolute configuration at this position. While returning HAT to the same position would likely recreate the stereogenic center with the same absolute configuration, spectroscopic and quantum chemical studies suggest that the hydrogen atom is delivered from the photocatalyst to a heteroatom that is in conjugation to the radical center. Two scenarios can be distinguished for the hydrogen atom shuttling process. For hydantoins, *N*-carboxyanhydrides, and 4,7-diaza-1-isoindolinones, the back HAT occurs to a carbonyl oxygen atom or an imine-type nitrogen atom which is not involved in binding to the catalyst. For oxindoles and 2,5-diketopiperazines, a single lactam carbonyl group in the substrate is available to accept the hydrogen atom. It is currently assumed that back HAT occurs to this group, although the carbonyl oxygen atom is involved in hydrogen bonding to the catalyst. In comparison to the former reaction pathway, the latter process appears to be less efficient and more prone to side reactions. For both cases, an achiral enol or enamine is formed, which delivers upon dissociation from the catalyst statistically either one of the two stereoisomers of the substrate. Since only one substrate enantiomer (or diastereoisomer) is processed, a high enantioselectivity (or diastereoselectivity) results. Even though the editing is a contra-thermodynamic process, the described decoupling of a photochemical and a thermal step allows the usage of a single catalyst in loadings that vary between 2.5 and 10 mol % depending on the specific mode of action.

## Key References

- GroßkopfJ.; PlazaM.; SeitzA.; BreitenlechnerS.; StorchG.; BachT.Photochemical Deracemization at sp^3^-Hybridized Carbon Centers via a Reversible Hydrogen Atom Transfer. J. Am. Chem. Soc.2021, 143, 21241–2124510.1021/jacs.1c1126634902253 .^1^ A chiral benzophenone catalyst was used to photochemically deracemize hydantoins in a highly enantioselective fashion. The discrimination of the two enantiomers by the chiral HAT catalyst was identified as a key event, but the mode of back HAT remained elusive.
- KuttaR. J.; GroßkopfJ.; van StaalduinenN.; SeitzA.; PrachtP.; BreitenlechnerS.; BannwarthC.; NuernbergerP.; BachT.Multifaceted View on the Mechanism of a Photochemical Deracemization Reaction. J. Am. Chem. Soc.2023, 145, 2354–236310.1021/jacs.2c1126536660908 .^2^ A suite of mechanistic experiments was employed to elucidate the detailed mechanism of hydantoin deracemization. The back HAT to an oxygen atom of the hydantoin was found to lead to an enol intermediate which tautomerizes unselectively.
- GroßkopfJ.; PlazaM.; KuttaR. J.; NuernbergerP.; BachT.Creating a Defined Chirality in Amino Acids and Cyclic Dipeptides by Photochemical Deracemization. Angew. Chem., Int. Ed.2023, 62, e20231360610.1002/anie.20231360637793026.^3^ 2,5-Diketopiperazines are cyclic dipeptides and belong to the most important amino acid derivatives. It was discovered that their photochemical deracemization by a chiral benzophenone was possible, although their carbonyl oxygen atom is involved in hydrogen bonding to the catalyst.

## 1 Introduction

Most chiral compounds display at least one, and frequently more than one, stereogenic carbon atom to which a hydrogen atom is attached. The importance of a defined absolute configuration in a chiral molecule becomes evident upon interaction with a chiral environment. Most notably, almost all biomolecules are intrinsically chiral and recognize the chirality of a possible binding partner. The lock-key model formulated by E. Fischer more than a century ago^4^ provides a catchy description for the recognition event between a drug and its biological target.

**Scheme 1:**
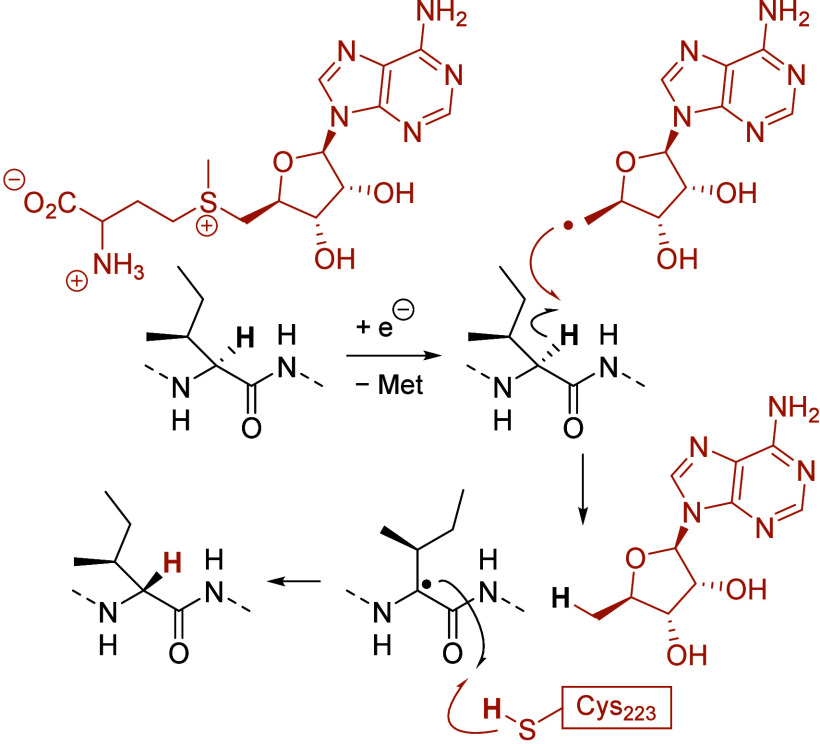
Stereochemical Editing by Nature: Epimerization of an Amino Acid within a Peptide Chain via a Radical Intermediate

When considering the adjustment of a stereogenic center in a given molecule, the reversible cleavage of a hydrogen atom represents a viable mechanistic pathway. A radical intermediate is formed in which the chiral information is erased and the stereogenic center can be recreated by hydrogen atom transfer (HAT) from an external reagent. Nature provides several impressive examples of how stereochemical editing by reversible HAT is accomplished. For example, a radical *S*-adenosyl-l-methionine (SAM) enzyme can invert the stereoinformation in the α-position of l-isoleucine and l-valine residues of a ribosomally synthesized peptide in *Bacillus subtilis*.^5^ YydG, the enzyme in question, is a two [4Fe-4S] cluster radical SAM enzyme whose SAM unit fragments (Met = methionine) upon single electron transfer from a [4Fe-4S]^+^ cluster.^6^ The generated 5′-deoxyadenosyl radical can then abstract a hydrogen atom of an amino acid residue of a peptide that fits into the active site of the enzyme, and back HAT from the opposite face occurs by a perfectly positioned cysteine (Cys) residue. The so-formed epipeptide YydF is a critical component in the bacterial cell envelope stress–response system (Scheme 1).^7^

**Figure 1:**
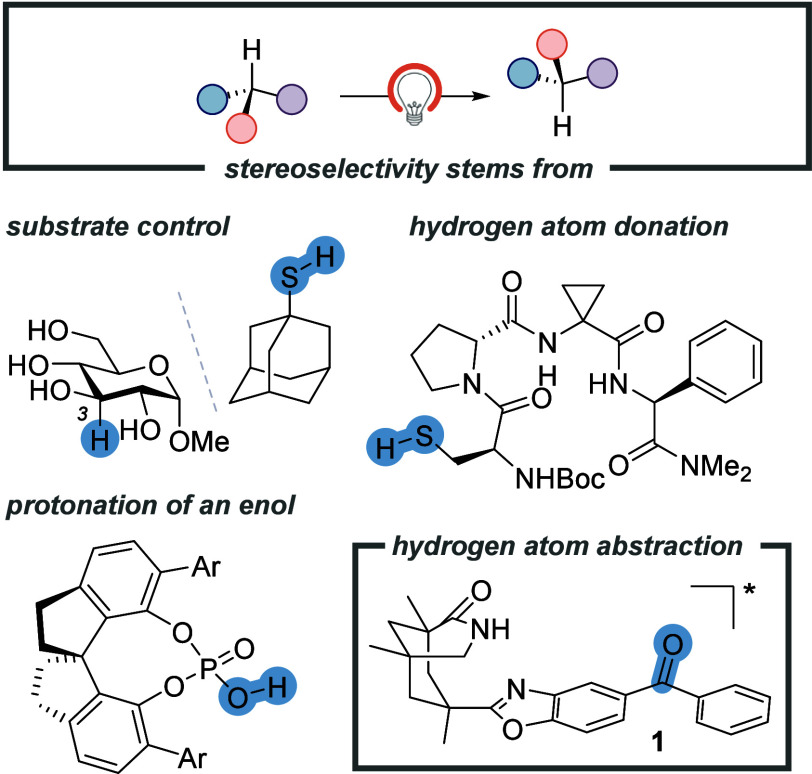
Overview of photocatalytic stereochemical editing processes based on reversible hydrogen atom transfer. Molecules with a single stereogenic center require chiral reagents to achieve photochemical deracemization.

In attempts to synthetically establish a stereogenic center by reversible HAT, the major distinction to be made relates to the three-dimensional structure of the substrate. If the compound displays more than one stereogenic center, then it is possible to tap on the intrinsic chirality of the molecular backbone to create the stereogenic center in a diastereoselective fashion. An illustrative example is the epimerization of carbohydrates as recently reported by the Wendlandt group.^8^ Here, the stereogenic center at carbon atom C3 of an α-methyl glucoside was adjusted by photochemically creating an amino radical cation, which is competent to remove the hydrogen atom at C3 by abstraction. Diastereoselective hydrogen atom donation by achiral 1-adamantanethiol leads to the formation of the respective α-methyl alloside (Figure 1).

If no other stereogenic center is present in the substrate, then reversible HAT can lead to a deracemization provided that the formation of the radical intermediate and the C–H bond forming event proceed in different reaction manifolds. Unlike diastereoisomers, enantiomers show identical scalar properties. In thermal equilibrium, both enantiomers are formed in equal amounts (racemate). Photochemistry offers a unique way to achieve deracemization^9,10^ by decoupling the creation of an achiral intermediate from the thermal formation of either one of the two enantiomers. In HAT chemistry, it is conceivable that the enantioselectivity is induced by a chiral hydrogen atom donor, such as a cysteine-derived peptidic thiol. Knowles, Miller, and co-workers employed the latter compound in combination with an enantioselective deprotonation of a photochemically generated radical cation for the deracemization of 4-substituted 3-aryl-2-imidazolidinones.^11^ Recent work by Ye and co-workers capitalized on the exclusive use of a chiral peptidic hydrogen atom donor for an enantioselective HAT.^12^ Alternatively, an unselectively generated radical can be reduced to an enol or enolate, which is protonated enantioselectively. Jiang and co-workers used a chiral spinol-derived phosphoric acid in combination with an achiral photoredox catalyst to achieve the photochemical deracemization of *N*-aryl-substituted α-amino acid esters.^13^

Our group had seen in photochemical deracemization reactions by triplet energy transfer that a single catalyst can successfully differentiate between two substrate enantiomers and facilitate an efficient deracemization.^14^ The observation led us to hypothesize that a similar strategy might be applicable to a reversible HAT process. Benzophenone **1** and its enantiomer *ent*-**1** turned out to be suitable and readily available^15^ catalyst systems, the applications of which will be presented in this Account. Beyond the synthetic results, mechanistic observations are discussed, and future directions will be mentioned. Procedures developed for photochemical deracemization are also applicable to compounds with multiple stereogenic centers. Typically, one center is selectively edited and allows for the preparation of a single diastereoisomer, which in turn opens several avenues for late-stage editing in advanced synthetic intermediates.

## 2 Substrates with an Unbound Oxygen Atom for Back HAT

Benzophenones can be excited by UV light in the wavelength region of λ = 330–380 nm. Although the nπ* transition is weak with absorption coefficients ε typically on the order of 100 M^–1^ cm^–1^, the subsequent steps are highly efficient. After promotion to the first singlet excited state S_1_, benzophenones benefit from a favorable spin–orbit coupling (El-Sayed rules)^16^ and undergo rapid intersystem crossing (ISC) to the lower-lying T_2_ state with ππ* character.^17^ The molecules reach the long-lived T_1_ state by internal conversion from T_2_. The electron deficiency at the oxygen atom in the nπ* triplet renders the T_1_ state highly reactive toward hydrogen atom abstraction. It has been reported that any C–H bond with a bond dissociation enthalpy below 450 kJ mol^–1^ (108 kcal mol^–1^) can be cleaved provided that the benzophenone oxygen atom is properly positioned.^18^ A distance to the hydrogen atom in the range of 230–310 pm has been suggested to guarantee a rapid abstraction.^19^

**Figure 2:**
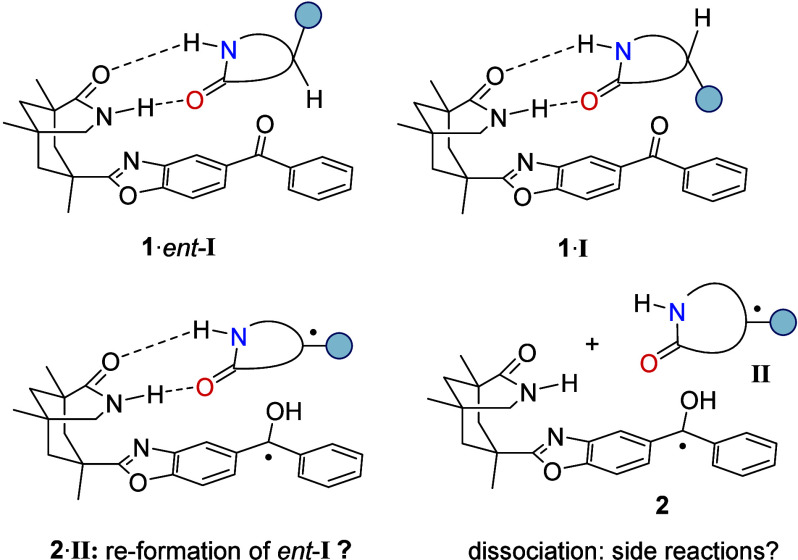
Association of the two enantiomers of generic chiral lactam *rac*-**I** to chiral benzophenone **1** (top) and possible consecutive pathways upon intramolecular HAT within complex **1**·*ent*-**I** (bottom).

Based on these fundamental considerations, the approach our group has taken to differentiate between two enantiomers by hydrogen abstraction was based on a putative hydrogen bonding between catalyst **1** and generic substrate *rac*-**I**. Without additional attractive forces, the association constant of a given lactam to benzophenone was found to be in the range of 100 M^–1^ (20 °C, benzene-*d*_6_).^2^ If the substrate displays a single stereogenic center within the lactam ring, then the hydrogen atom is properly positioned within complex **1**·*ent*-**I** for hydrogen atom abstraction by photoexcited benzophenone. The latter arrangement can be considered a matched situation, as opposed to the mismatched complex **1**·**I** in which intramolecular HAT at the stereogenic center is difficult to achieve (Figure 2).

While the HAT within complex **1**·*ent*-**I** leads to protonated ketyl radical **2** and the substrate radical **II**, a productive deracemization requires the back HAT to lead not only to *ent*-**I** but also to enantiomer **I**. If the hydrogen atom was transferred directly to the prostereogenic radical center within complex **2**·**II**, then the same enantiomer would likely be reformed. If the molecules dissociated to the individual radicals **2** and **II**, then an unselective back HAT would be possible. However, given the high reactivity of radicals, side reactions should result. In this regard, a key challenge for a deracemization based on the described approach is the quest for a reliable back HAT.

### 2.1 Hydantoins

**Scheme 2:**
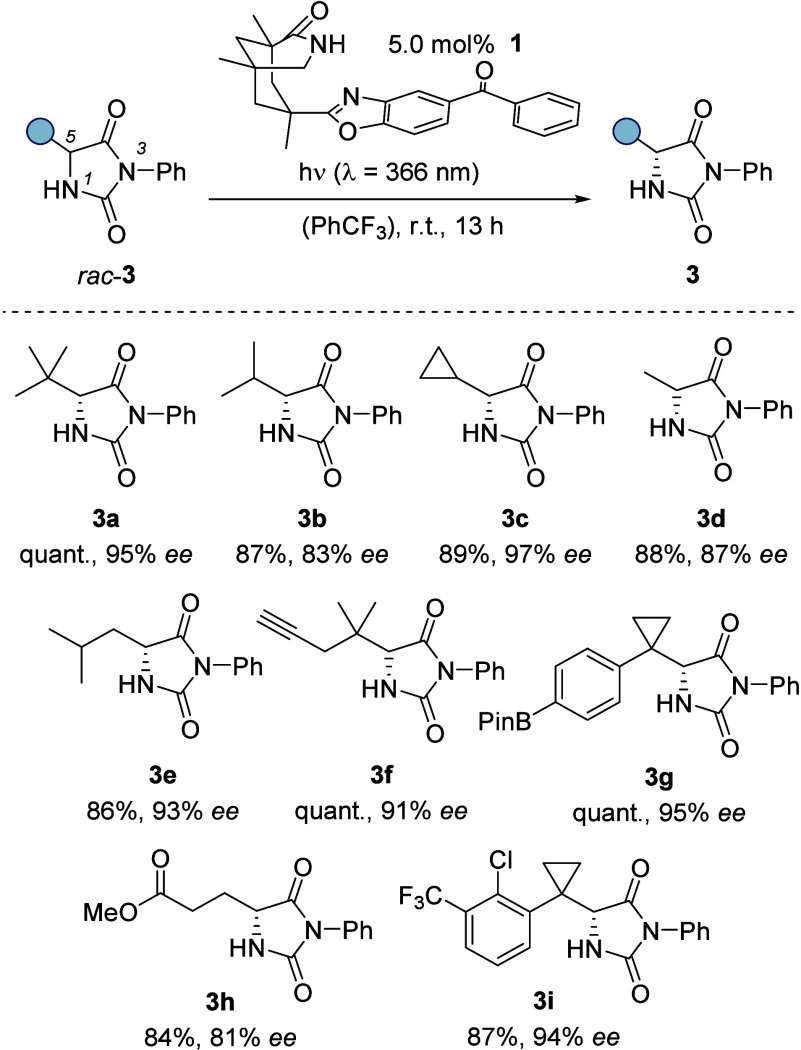
Catalytic Photochemical Deracemization of Imidazolidine-2,4-diones (Hydantoins) *rac*-**3**: Reaction Conditions and Representative Products

Imidazolidine-2,4-diones (hydantoins) are cyclic α-amino acid derivatives that are known to be configurationally stable and that are accessible in racemic form by a number of methods. The stereogenic center at the 5-position invited a possible deracemization with the free NH at position N1 and the carbonyl group at C2 providing a coordination motif for hydrogen bonding to catalyst **1**. The substituent at nitrogen atom N3 can be freely chosen, and it was found that a phenyl group provides the highest enantioselectivity. A catalyst loading of 5 mol % was sufficient to allow for an efficient deracemization as shown by 26 examples.^1^ The best results were recorded for substrates *rac*-**3** with secondary or tertiary alkyl groups attached to carbon atom C5. The deracemization protocol was compatible with olefin, alkyne, nitrile, ester, ether, and boronate groups as well as with halogen substituents fluorine, chlorine, and bromine. The major enantiomers **3** were shown to be (*R*)-configured at the stereogenic carbon atom C5 (Scheme 2, *ee* = enantiomeric excess, Pin = pinacolato).

**Scheme 3:**
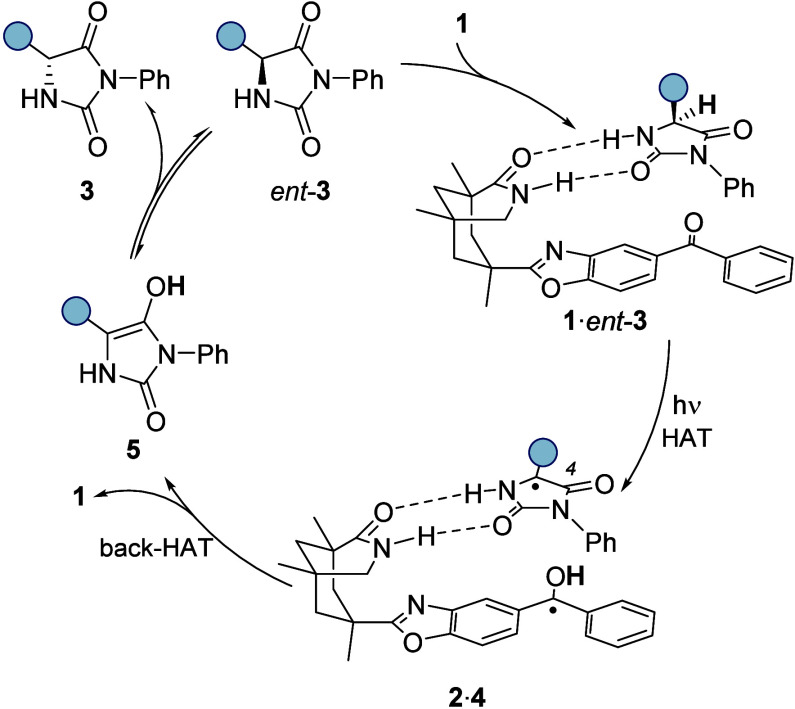
Suggested Mechanism for the Catalytic Photochemical Deracemization of Hydantoins *rac*-**3**

Labeling studies indicated scrambling of deuterium from deuterated to undeuterated substrates in the course of the reaction ruling out an immediate back HAT within a substrate-catalyst complex (Figure 2). In stark contrast to the reaction of hydantions *rac*-**3** and parent benzophenone (PhCOPh), which led to side products from radical–radical coupling processes, the photoderacemization was remarkably clean, and there was no indication of side reactions. The difference in association of the two hydantoin enantiomers **3i** and *ent*-**3i** to catalyst **1** was relatively small (K_a_ = 89 M^–1^ in a matched vs K_a_ = 56 M^–1^ in a mismatched situation). Eventually, a combination of spectroscopic and computational methods revealed that an enol intermediate was involved in the deracemization reaction. Transient absorption spectroscopy was performed by employing compounds **3i** and *ent*-**3i** as substrates. Spectroscopic evidence for an enol was obtained by a transient signal with an absorption of around λ ≅ 330 nm and a lifetime of 2 μs.^2^ Computational studies suggested rapid hydrogen bond formation of the protonated ketyl radical **2** to the carbonyl oxygen atom at C4 of hydantoin-derived radical **4** followed by a back HAT to this atom. Subsequent tautomerization of enol **5** likely occurs via a mutual proton exchange in a dimer^20^ or by an intermolecular protonation/deprotonation mechanism accounting for the observed deuterium scrambling (Scheme 3).

**Figure 3:**
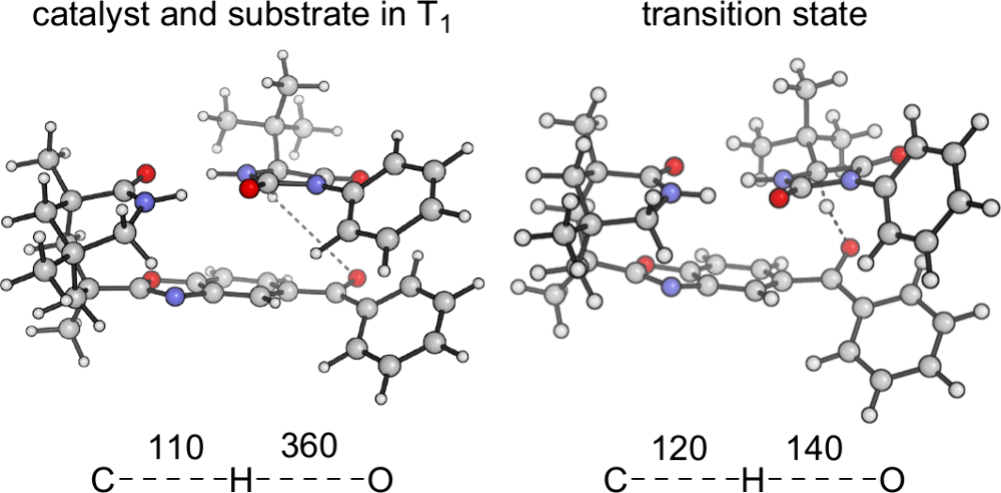
Calculated distances (all values in pm) within the complex of catalyst **1** and substrate *ent*-**3a** in the triplet state and in the transition state.

The lifetime of triplet excited benzophenone **1** was shortened upon addition of the matched substrate from 206 to 181 ns (aerobic conditions), suggesting the HAT to occur with a quantum yield of Φ = 0.12. Protonated ketyl radical **2** was identified by its transient signal showing a red-shifted (λ ≅ 550 nm) absorption relative to the triplet benzophenone that peaks at λ = 525 nm. The rate for back HAT is high, with only small signatures of the radical **2** being detected by transient absorption spectroscopy. The spectral features of the counter radical **4** were not observed, which is in line with calculations suggesting that it displays a significantly lower extinction coefficient than radical **2**. In the most stable conformation, the distance between the carbonyl oxygen atom of the catalyst to the hydrogen atom at the stereogenic center was calculated to be 360 pm (Figure 3) for complex **1**·*ent*-**3a**. Rotation around the benzoxazole bond to the azabicyclo[3.3.1]nonan-2-one leads to a conformation with an even smaller distance. In the former arrangement, a transition state was identified for the HAT with almost equal distances of the hydrogen atom to the two heavier atoms. When hydrogen and deuterium are compared, the calculated HAT reaction barrier increases from 57.0 kJ mol^–1^ (for hydrogen) to 58.6 kJ mol^–1^ (for deuterium). The calculated kinetic isotope effect *k*_H_/*k*_D_ of 1.9 was in good agreement with the experimental value which was found to be 1.4 for the reaction of hydantoin *ent*-**3e**.

The somewhat incoherent results obtained with seemingly similar substrates such as *rac*-**3b** and *rac*-**3c** (Scheme 3) are likely related to the fact that a HAT can occur from other positions within the substrate to the photoexcited catalyst. Catalyst decomposition results, which slows down the conversion and leads to an incomplete deracemization.

### 2.2 *N*-Carboxyanhydrides

**Scheme 4:**
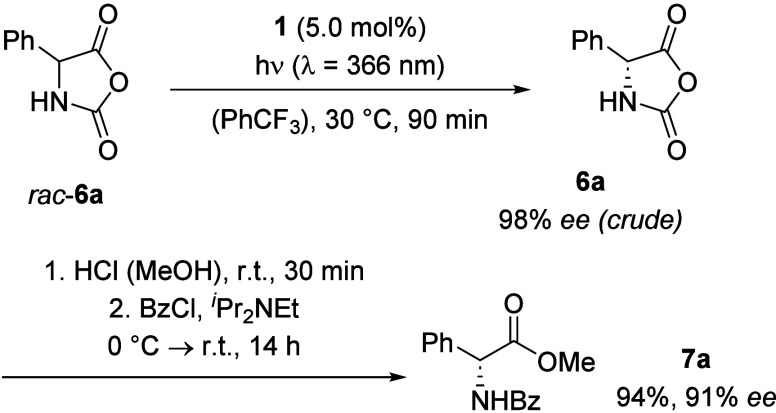
Photochemical Deracemization of Phg-NCA (*rac*-**6a**) and Conversion to Its *N*-Benzoylated Methyl Ester **7a**

Due to their structural similarity to hydantoins, *N*-carboxyanhydrides (NCAs) of α-amino acids emerged as potential new substrates for the established deracemization protocol. Swapping the *N*-phenyl group in the 3-position with an oxygen atom leads to a significantly lower stability of NCAs compared to hydantoins.^21^ While hydantoins are configurationally stable and can be converted to their parent amino acids only under strongly basic or acidic conditions at elevated temperatures,^22,23^ NCAs display a high instability toward moisture and racemize in protic solvents or in the presence of a nucleophile/base under ambient conditions.^24^ On the one hand, the high lability severely complicates quantifying the yield and enantioselectivity of an NCA deracemization. On the other hand, it also provides a handle for a possible deracemization–derivatization sequence of a racemic amino acid, especially since NCAs can be obtained in a single-step condensation reaction from an amino acid with triphosgene requiring no further purification. Hence, after promising initial deracemization results, a reliable and reproducible method to convert the deracemized NCAs into stable and easy-to-analyze amino acid derivatives was needed. Deracemization of phenylglycine (Phg)-derived NCA (*rac*-**6a**) with catalyst **1** resulted in 98% *ee* after only 90 min, and an extensive methanolysis study on phenylglycine-derived NCA showed that increasingly more acidic environments led to higher degrees of stereoretention and full conversion for the formation of phenylglycine methyl ester hydrochloride under ambient conditions within a few minutes. As an additional step, the free amine was benzoylated to product **7a**, ensuring a reliable *ee* determination by chiral HPLC (Scheme 4).^25^

**Scheme 5:**
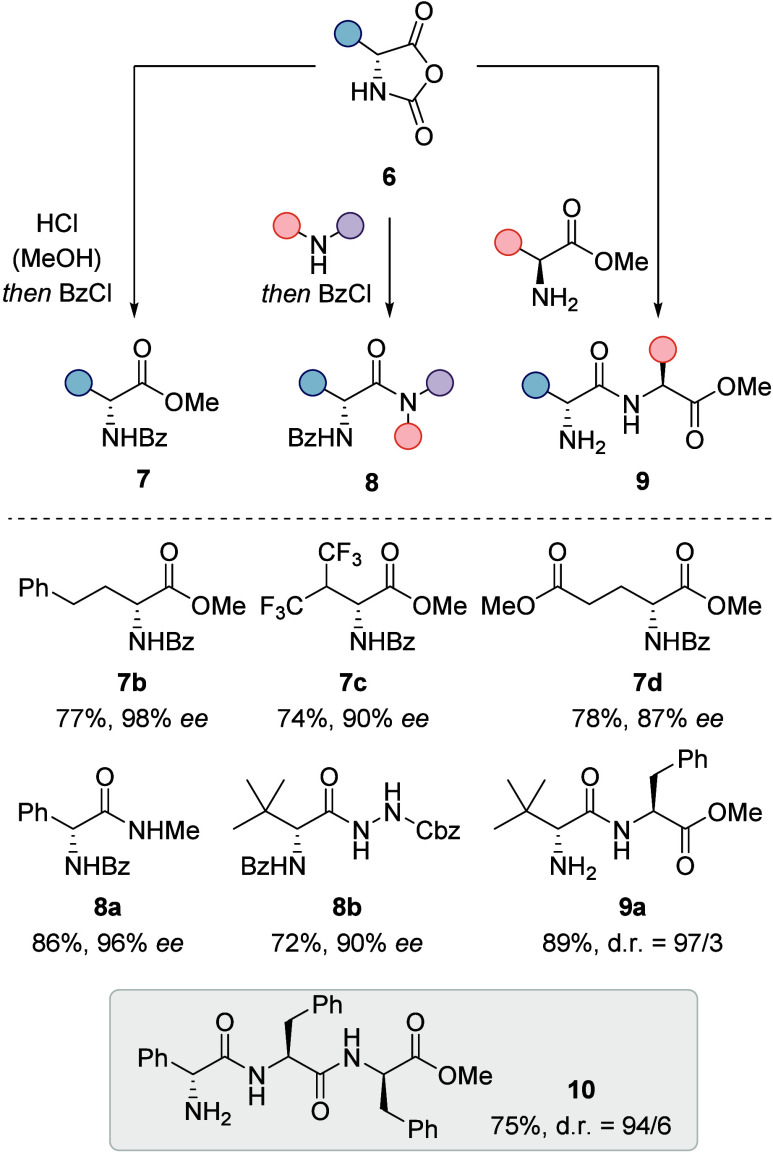
Downstream Reactions of Deracemized NCAs **6** and a Selection of Examples for the Different Accessible Derivatives

Other NCAs performed worse in the deracemization reaction, and catalyst decomposition was identified as the main reason. To ensure the best possible results, the standard reaction time was increased to 5 h with a catalyst loading of 5.0 mol %. In most cases, enantioselectivities could then be improved by adding another portion of **1** (5.0 mol %) followed by another 5 h of irradiation time. A deracemization mechanism can be invoked that is similar to the mechanism established for hydantoins (Scheme 3), and the absolute configuration of the obtained products was expectedly identified to be the (*R*)-configuration. Functional group tolerance encompassed esters, halogen substituents, ethers, and trifluoromethyl groups apart from the intrinsically existing carbonyl and amide groups. In total, 14 amino acid methyl esters were obtained with enantioselectivities exceeding 82% *ee*. To highlight the synthetic potential of the method, NCAs **6** were converted to amides (**8**) with an array of different amines (9 total examples). Additionally, the synthesis of two dipeptides (e.g. **9a**) and one tripeptide (**10**) using enantioenriched NCAs as activated coupling reagents underlines their usefulness in peptide chemistry (Scheme 5).

**Scheme 6:**
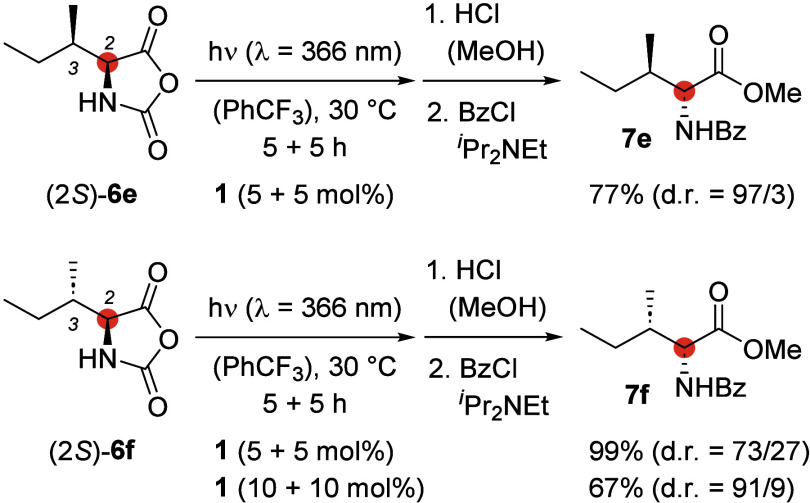
Stereochemical Editing: Selective Inversion of Stereogenic Center C2 of l-Allo-isoleucine [(2*S*)-**6e**] and l-Isoleucine [(2*S*)-**6f**]

Since the proposed deracemization mechanism requires an intermediate enol, exclusive stereochemical editing of the stereogenic center in the 2-position of l-allo-isoleucine [(2*S*)-**6e**] and l-isoleucine [(2*S*)-**6f**] appeared viable while the stereoinformation in the 3-position was expected to remain intact. Indeed, the developed protocol showed almost complete stereoinversion at carbon atom C2 and no change at stereogenic center C3 for both amino acids, even though, for l-isoleucine, a higher catalyst loading was necessary (Scheme 6) to achieve a high diastereomeric ratio (d.r.).

## 3 Substrates with a Hydrogen-Bonded Oxygen Atom for Back HAT

### 3.1 Oxindoles

**Scheme 7:**
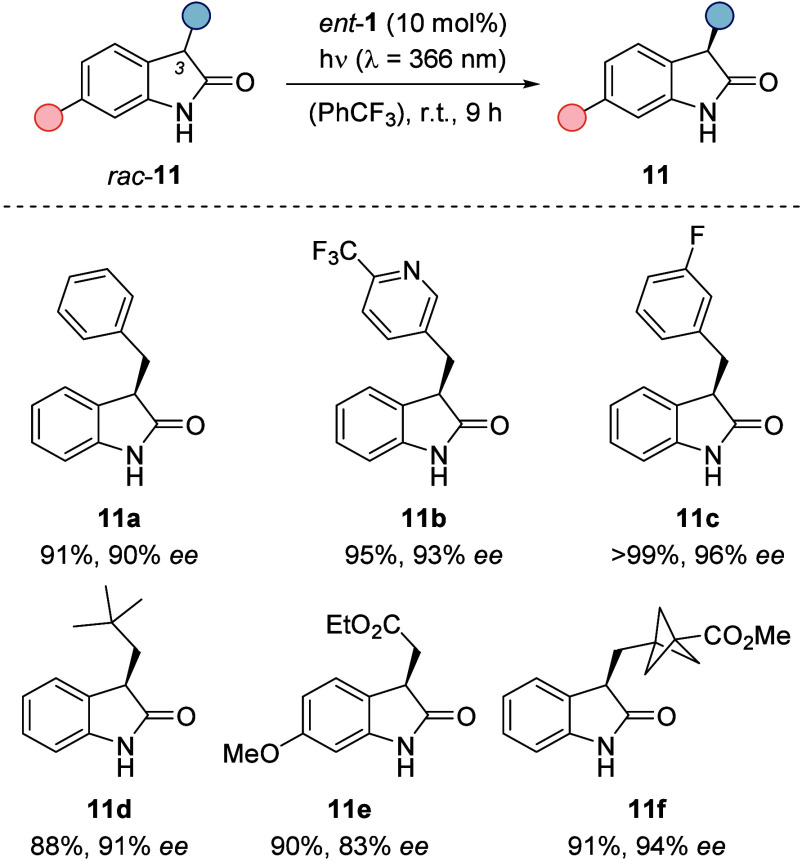
Catalytic Photochemical Deracemization of 2-Indolinones (Oxindoles) *rac*-**11**: Reaction Conditions and Representative Products

2-Indolinones (oxindoles) comprise an important class of heterocyclic compounds and show a wide range of biological activities.^26−28^ While 3,3-disubstituted oxindoles can be synthesized enantioselectively using conventional methods,^29^ the enantioselective synthesis of 3-monosubsituted oxindoles has long remained elusive due to their high C–H acidity in the 3-position. A deracemization protocol promised to provide access to enantioenriched oxindoles, since mild conditions are applied in a nonpolar solvent. As established for hydantoins **3**, the prerequisite for successful deracemization is a smooth back HAT, which for hydantoins occurs to the carbonyl oxygen atom that is not hydrogen-bonded to catalyst **1** (Scheme 3). Since oxindoles possess only one carbonyl group, back HAT would have to occur to the hydrogen-bonded oxygen atom to enable a successful deracemization. Gratifyingly, it was found that a variety of racemic 3-substituted oxindoles (*rac*-**11**) could indeed be deracemized using catalyst *ent*-**1**, the enantiomer of the previously used photocatalyst **1** (Scheme 7).^30^ Trifluorotoluene was confirmed to be the superior solvent, and conditions were optimized to a catalyst loading of 10 mol % and an irradiation time of 9 h. Esters, ethers, halides, pyridines, and a strained bicyclo[1.1.1]pentane were tolerated as functional groups, and in total, 24 oxindoles were deracemized with enantioselectivities ranging from 80 to 99% *ee*. The absolute configuration of the deracemized product **11** was identified to be (*R*), which is in accordance with the assumed favored hydrogen atom abstraction of substrate enantiomer (*S*)-**11** by photocatalyst *ent*-**1**. To further investigate the nature of the back HAT in this deracemization, a deuterium scrambling experiment was conducted which showed that deuterium was transferred from a fully deuterated substrate to a nondeuterated substrate in the photoreaction. The result is in line with a keto–enol tautomerization, suggesting the existence of an intermediate enol and therefore back HAT to the hydrogen-bonded carbonyl oxygen.

**Scheme 8:**
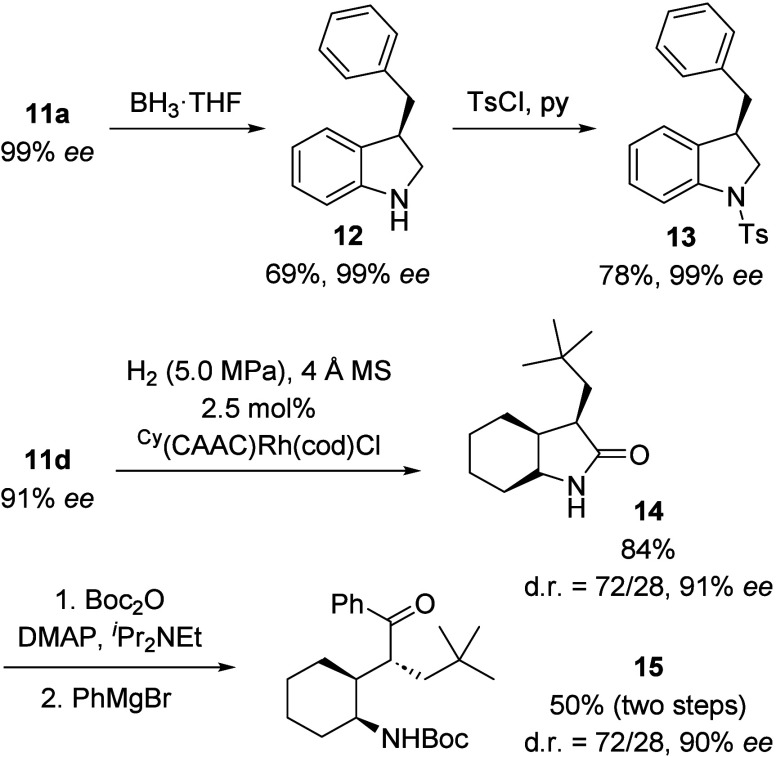
Examples for Downstream Chemistry of Enantioenriched Oxindoles **11** and Accessible Derivatives **12**–**15** ^Cy^(CAAC) = cyclic (alkyl)-(amino)carbene; cod = 1,5-cyclooctadiene; DMAP = 4-(dimethylamino)pyridine; MS = molecular sieve; py = pyridine; Ts = *p*-toluenesulfonyl.

To showcase the potential applications of oxindole deracemization, some photoproducts were further derivatized. For example, oxindole **11a** (99% *ee*) was reduced to indoline **12** and further *N*-tosylated to product **13** with no loss of enantiopurity. Oxindole **11d** was fully hydrogenated to hexahydrooxindole **14** and then converted to *N*-*tert*-butyloxycarbonyl(Boc)-protected aminoketone **15** with three contiguous stereogenic centers. The absolute configuration was retained (Scheme 8).

### 3.2 2,5-Diketopiperazines

**Scheme 9:**
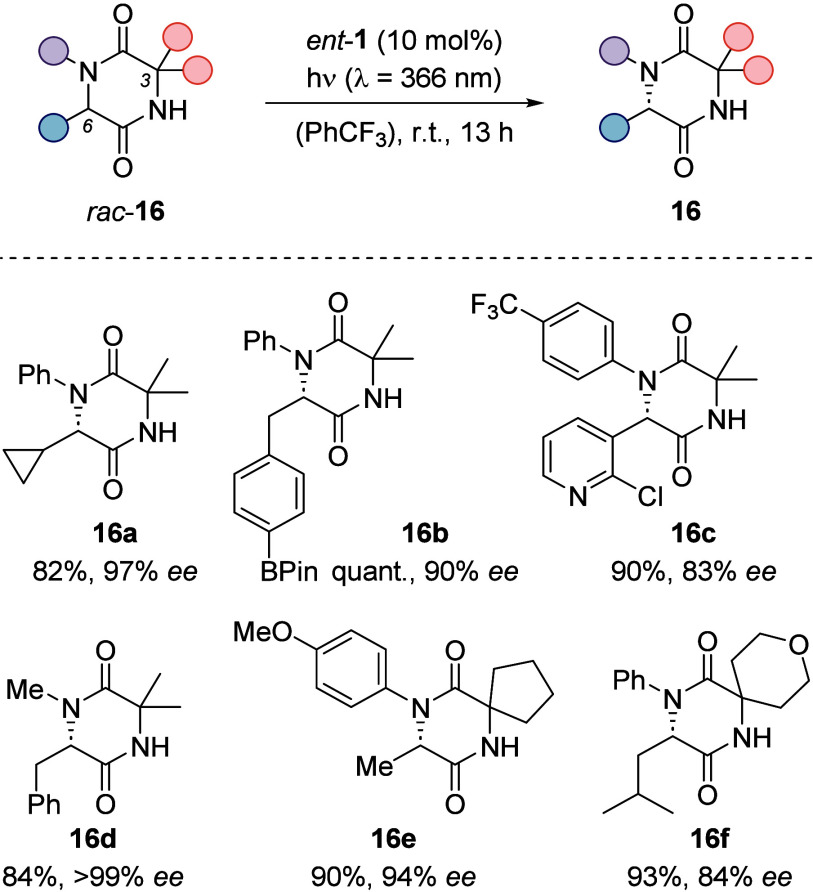
Catalytic Photochemical Deracemization of 2,5-Diketopiperazines *rac*-**16**: Reaction Conditions and Representative Products

2,5-Diketopiperazines are cyclic dipeptides, and they present another intriguing substrate class for photochemical deracemization. They can be used as precursors for *N*-substituted amino acids and display a wide range of pharmacological activities.^31^ Racemic 2,5-diketopiperazines (*rac*-**16**) were prepared by an Ugi four-component reaction and were amenable to a deracemization protocol with catalyst *ent*-**1**.^3^ High enantioselectivities were obtained with different substitutions at C3 and with both aryl and alkyl substituents at N1. In addition, esters, ethers, halides, boronates, alkynes, and pyridines were tolerated as functional groups, confirming their resistance toward the photoexcited benzophenone. A comparison with authentic amino acids after hydrolysis revealed the deracemized products **16** to display an (*S*)-configuration. Groups that would undergo oxidation with the photoexcited benzophenones, or hydrogen atoms, other than the one at C6, that could be easily abstracted by the photocatalyst hampered a smooth deracemization. With no back HAT motif available, protonated ketyl radical **2**/*ent*-**2** (Scheme 3) is prone to undergo side reactions, e.g., radical–radical coupling, consequently leading to catalyst decomposition. In addition, as indicated by NOESY-NMR experiments, bulky substituents at C6 forced 2,5-diketopiperazines to adopt a boat-like instead of a planar conformation, which in turn provided an unfavorable trajectory for hydrogen atom abstraction by the photocatalyst. In total, 53 2,5-diketopiperazines were successfully deracemized with enantioselectivites ranging from 71 to 99% *ee* (Scheme 9).

**Scheme 10:**
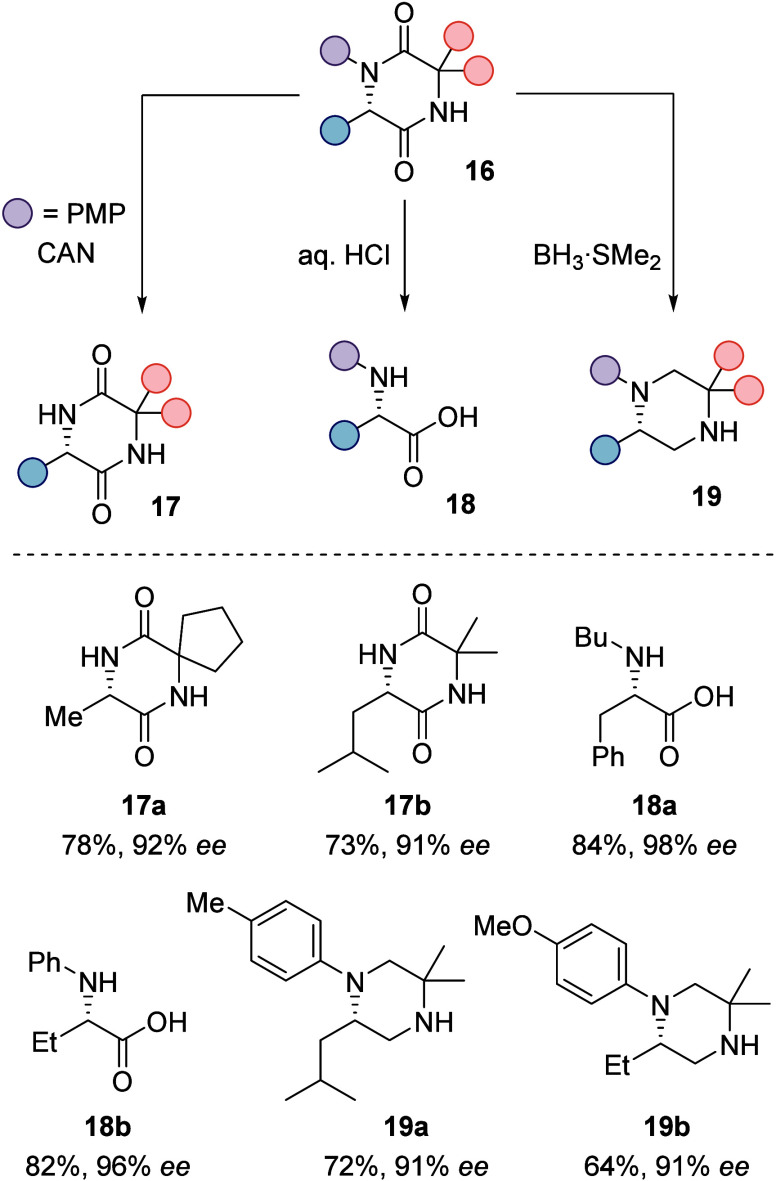
Examples for Downstream Chemistry for Enantioenriched 2,5-Diketopiperazines and Accessible Derivatives **17**–**19**

The high synthetic potential of 2,5-diketopiperazines was illustrated by several downstream reactions, leading to a variety of amino acid derivatives. For example, if N1 was connected to a *para*-methoxyphenyl (PMP) group, then treatment with ceric ammonium nitrate (CAN) generated unprotected 2,5-diketopiperazines **17** with two free NH groups. Acidic hydrolysis of the enantioenriched photoproducts furnished in total ten *N*-aryl- or *N*-alkyl-substituted amino acids **18**, and fully saturated piperazines **19** were obtained from the reduction of 2,5-diketopiperazines with the borane dimethylsulfide adduct. All transformations proceeded smoothly with no or only little loss of enantiopurity (Scheme 10).

**Scheme 11:**
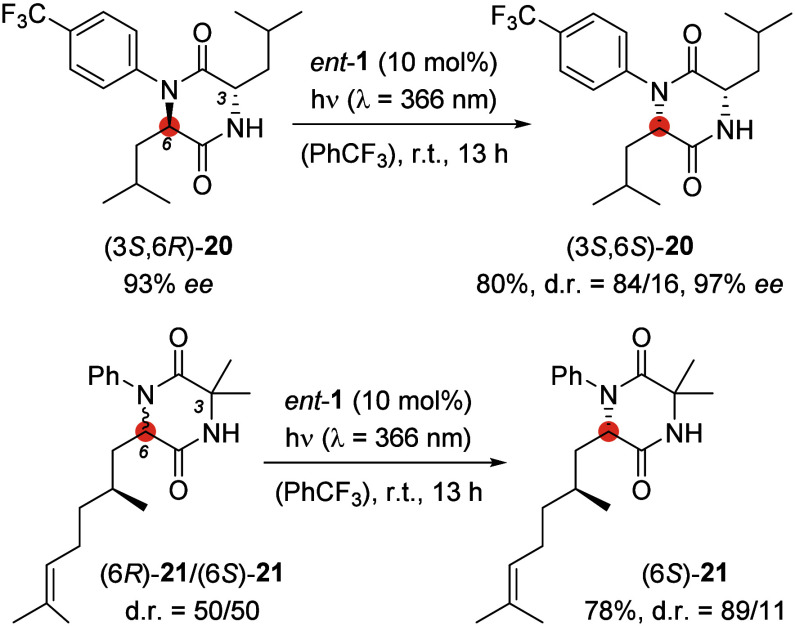
Stereochemical Editing: Examples for Selective Editing of the Stereoinformation at C6 for 2,5-Diketopiperazines **20** and **21** with More Than One Stereogenic Center

Finally, it was shown that the established deracemization protocol was suitable for the stereochemical editing of only the stereogenic center at C6, while other stereogenic centers remained untouched. 2,5-Diketopiperazine (3*S*,6*R*)-**20** is a leucine dimer with two different stereogenic centers. Catalyst *ent*-**1** in its triplet state can abstract only the hydrogen atom at the stereogenic center with the (6*R*)-configuration but not at the one with the (3*S*)-configuration; consequently, the stereoinformation at C3 remained intact while the one at C6 got inverted to furnish product (3*S*,6*S*)-**20** with a d.r. of 84/16 and an *ee* of 97%. Irradiation of a diastereomeric mixture of substrate (6*R*,6*S*)-**21** in the presence of photocatalyst *ent*-**1** allowed for the selective stereochemical editing in the 6-position while the stereoinformation at the alkyl substituent attached to C6 stayed intact. Product (6*S*)-**21** was obtained in a d.r. of 89/11 (Scheme 11).

**Figure 4:**
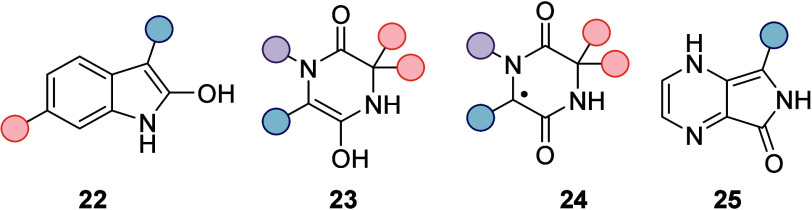
Possible intermediates **22**–**24** in the deracemization of oxindoles *rac*-**11** and diketopiperazines *rac*-**16**. Enamine **25** is a putative intermediate formed by back HAT to a nitrogen atom.

As briefly mentioned in this and the previous section, it was speculated that back HAT for both oxindoles and 2,5-diketopiperazines would occur to the hydrogen-bonded oxygen atom, resulting in enols **22** and **23**. Attempts to detect enol **23** by transient absorption spectroscopy remained futile, however. It could be validated for the diketopiperazine deracemization that the minor enantiomer *ent*-**16** undergoes a more efficient HAT than the major enantiomer **16**, but transient signals on a longer time scale could not be assigned to the enol. Rather, a long-lived protonated ketyl radical *ent*-**2** (cf. Figure 2) seemed to persist that was associated with either the diketopiperazine radical **24** or a substrate molecule (Figure 4).

Experimentally, it was obvious that substrates *rac*-**11** and *rac*-**16**, for which a back HAT required the oxygen atom involved in hydrogen bonding to the catalyst, reacted less efficiently. The catalyst loading was 10 mol %, indicating decomposition of the catalyst to occur. In a search for substrates in which back HAT would be smooth, we envisioned an enamine species **25** to be a possible intermediate that would be formed from 4,7-diaza-1-isoindolinones by back HAT to a nitrogen atom.

## 4 Substrates with a Nitrogen Atom for Back HAT: 4,7-Diaza-1-isoindolinones

**Scheme 12:**
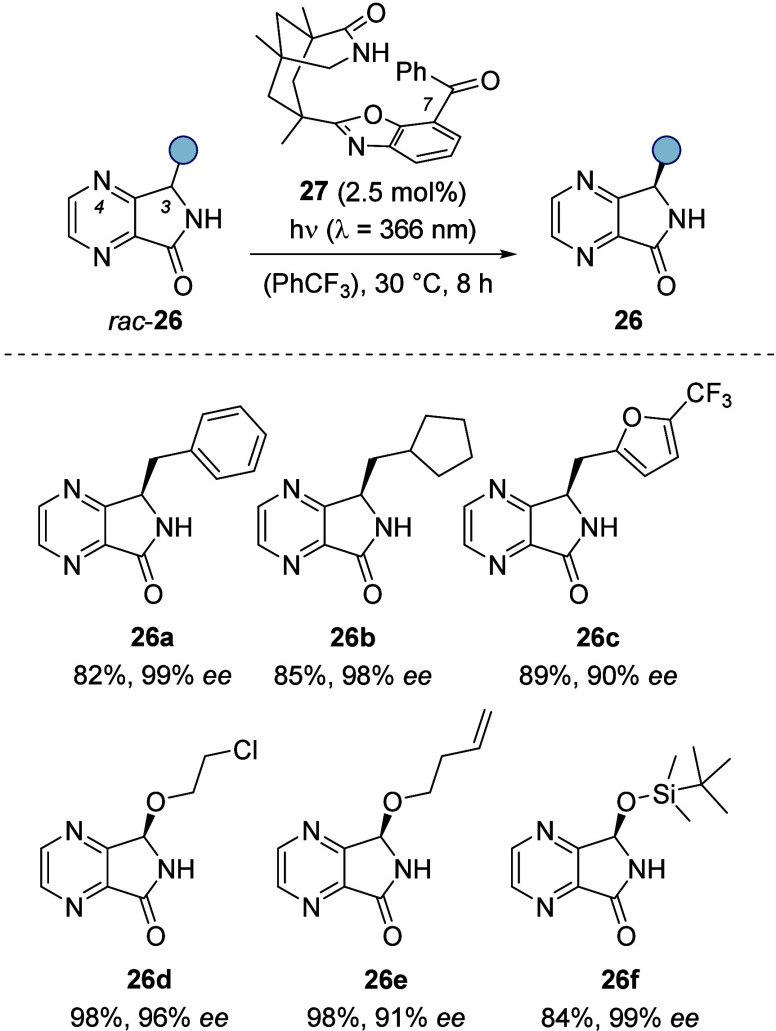
Catalytic Photochemical Deracemization of 4,7-Diaza-1-isoindolinones *rac*-**26**: Reaction Conditions and Representative Products

4,7-Diaza-1-isoindolinones display not only a hydrogen-bonding motif suitable for recognition by azabicyclo[3.3.1]nonan-2-one-based catalysts but also exhibit an imine nitrogen atom within the heterocyclic core that seemed suitable for back HAT from a protonated ketyl radical. Initial considerations of the relative topology of the 3-position of 4,7-diaza-1-isoindolinones (*rac*-**26**) and the photoactive benzophenone unit of the catalyst suggested compound **27** (benzophenone in the 7-position of the benzoxazole ring) to be a more promising catalyst for this deracemization than compound **1** (benzophenone in the 5-position of the benzoxazole). Indeed, the deracemization with benzophenone **27** proceeded smoothly at very low catalyst loadings (2.5 mol %) and enabled the preparation of a broad variety of products **26** with excellent enantioselectivities (Scheme 12).^32^

Irradiation of racemate *rac*-**26a** in the presence of catalyst **1** led to negligible enantioselectivity (2% *ee*). For some previous examples (*vide supra*), catalyst decomposition due to an inefficient back HAT necessitated higher catalyst loadings. In contrast, the low catalyst loading required for the deracemization of 4,7-diaza-1-indolinones suggests a very efficient back HAT in this case. H/D scrambling experiments as well as computational studies support the existence of intermediate enamine **25** (Figure 4) which is formed by returning HAT from the protonated ketyl radical of catalyst **27** to N4 of 4,7-diaza-1-isoindolinones. The process is initiated by a recognition event in which one enantiomer of the substrate undergoes selective HAT to the benzophenone catalyst, as outlined for hydantoins (Scheme 3). In line with our expectations, the respective (*S*)-isomer was processed while the deracemization products were found to display the (*R*)-configuration. Halides, ethers, esters, alkenes, furans, and silyl-protected alcohols were tolerated as functional groups. It is especially noteworthy that enantioenriched 3-oxygen-substituted 4,7-diaza-1-isoindolinones (**26d**–**f**) were accessible with this method since the corresponding hydroxy compound is an *N*,*O*-hemiacetal and racemizes readily.^33^

**Scheme 13:**
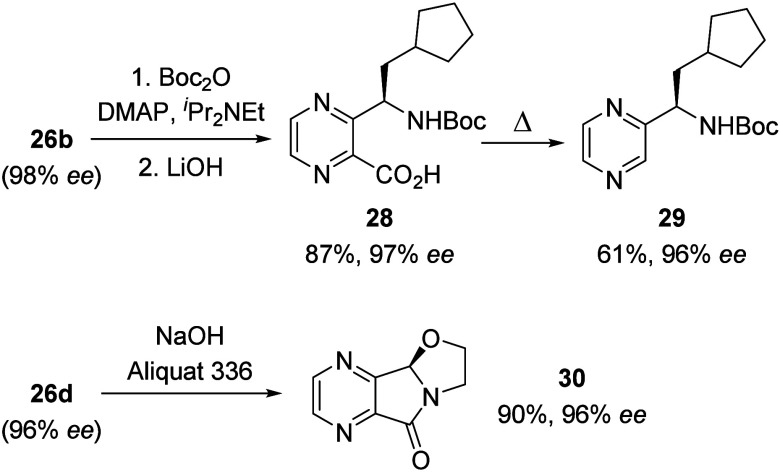
Examples for Downstream Chemistry of Enantioenriched 4,7-Diaza-1-isoindolinones **26b** and **26d** Leading to Pyrazine Derivatives **28**–**30**

Further downstream chemistry of the enantioenriched 4,7-diaza-1-isoindolinones included, for example, Boc protection of the lactam nitrogen atom of product **26b** followed by saponification. The reaction sequence gave access to ring-opened carboxylic acid **28**, the *ee* of which was determined via its methyl ester and which could then be further decarboxylated to pyrazine **29** bearing a stereogenic center in the α-position (Scheme 13).

Chloroethyl-substituted isoindolinone **26d** underwent an intramolecular alkylation reaction with the lactam nitrogen atom to form oxazolidine **30** with sodium hydroxide under phase-transfer conditions.

## 5 Conclusions

The fact that a single chiral catalyst can induce an efficient deracemization appears at first sight to be counterintuitive. However, issues of microscopic reversibility can be overcome if a photochemical step is decoupled from a subsequent thermal event. The presented strategy involves a chiral photocatalyst that processes exclusively a single enantiomer and induces its racemization. The photostationary state is out of thermal equilibrium because the other enantiomer is continuously formed without being consumed in the racemization process. Although we are convinced that the concept is of general value and holds promise for many further applications, we also foresee cases for which additives might have a beneficial effect on the photocatalytic performance.
